# Targeting myeloid cells to improve cancer immune therapy

**DOI:** 10.3389/fimmu.2025.1623436

**Published:** 2025-07-31

**Authors:** Hui Chen, Zihan Xu, Judith Varner

**Affiliations:** ^1^ Moores Cancer Center, University of California, San Diego, La Jolla, CA, United States; ^2^ Department of Pathology, University of California, San Diego, La Jolla, CA, United States

**Keywords:** myeloid cell, tumor associated macrophage (TAM), PI3Kgamma, TREM2, CSF1R (colony stimulating factor 1 receptor), Axl, LILRB, immunesuppression

## Abstract

Tumor immunosuppression remains a major barrier to effective cancer immunotherapy and is often driven by the immunoregulatory activities of innate immune cells, such as myeloid cells within the tumor microenvironment (TME). Myeloid populations—including tumor-associated macrophages (TAMs), dendritic cells, granulocytes, monocytes and myeloid-derived suppressor cells (MDSCs)—play pivotal roles in dampening anti-tumor immune responses and promoting tumor progression. Recent advances in our understanding of myeloid cell biology have unveiled new therapeutic opportunities to disrupt these immunosuppressive mechanisms associated with tumor inflammation. This review highlights key signaling pathways and surface molecules involved in myeloid-mediated immune suppression, including CSF1R, PI3Kγ, mTOR, Syk, MerTK/Axl, and immune checkpoints such as Trem2, LILRBs, VISTA, and CD40. We examine preclinical and clinical findings that support targeting these pathways to reprogram the TME and enhance anti-tumor immunity. By integrating insights from mechanistic studies and therapeutic development, this review underscores the potential of myeloid cell-targeting strategies as promising adjuncts to current cancer immunotherapies. Finally, we discuss future directions and challenges in translating these approaches into durable clinical benefit.

## 1 Introduction

Tumor immunosuppression is a hallmark of cancer that enables malignant cells to evade immune surveillance and sustain unchecked growth (1). Within the tumor microenvironment (TME), cancer cells orchestrate a complex network of immunosuppressive mechanisms that impair both innate and adaptive immune responses. As a consequence, immunosuppression facilitates tumor progression by creating an environment that supports cancer cell survival, proliferation, angiogenesis, and metastasis. It also presents a major obstacle to effective cancer immunotherapy (2, 3). Although immune checkpoint inhibitors have revolutionized treatment for several cancers, their efficacy is often limited by the extent of baseline or acquired immunosuppression in the TME (4). Targeting immunosuppressive pathways holds promise not only for restoring anti-tumor immunity but also for improving long-term outcomes in cancer patients.

Myeloid cells—including macrophages, dendritic cells (DCs), and granulocytes, play critical and multifaceted roles in shaping the TME. These cells, derived from hematopoietic progenitors, are key regulators of immune responses and tissue homeostasis. Consequently, targeting myeloid cells and their associated signaling pathways has emerged as a promising strategy in cancer immunotherapy, aiming to reprogram or deplete the immunosuppressive subsets while enhancing pro-inflammatory and antigen-presenting functions. Myeloid cells are central players in the immunosuppressive landscape of the TME. These cells can be co-opted by tumors to promote immune tolerance and inhibit anti-tumor immunity through a variety of mechanisms. The plasticity and adaptability of myeloid cells allow tumors to shape their function dynamically, presenting significant challenges—but also therapeutic opportunities—in cancer treatment. Overcoming myeloid-mediated immune evasion has become a critical focus in the advancement of effective cancer immunotherapy. Myeloid cells not only inhibit anti-tumor immune responses through the secretion of immunosuppressive cytokines and metabolic factors, but also express immune checkpoint molecules such as PD-L1, which directly suppress T cell function (5–8). As a result, the presence of immunosuppressive myeloid cells within the TME is strongly associated with poor prognosis and resistance to immune checkpoint blockade therapies in multiple cancer types.

**Figure 1:**
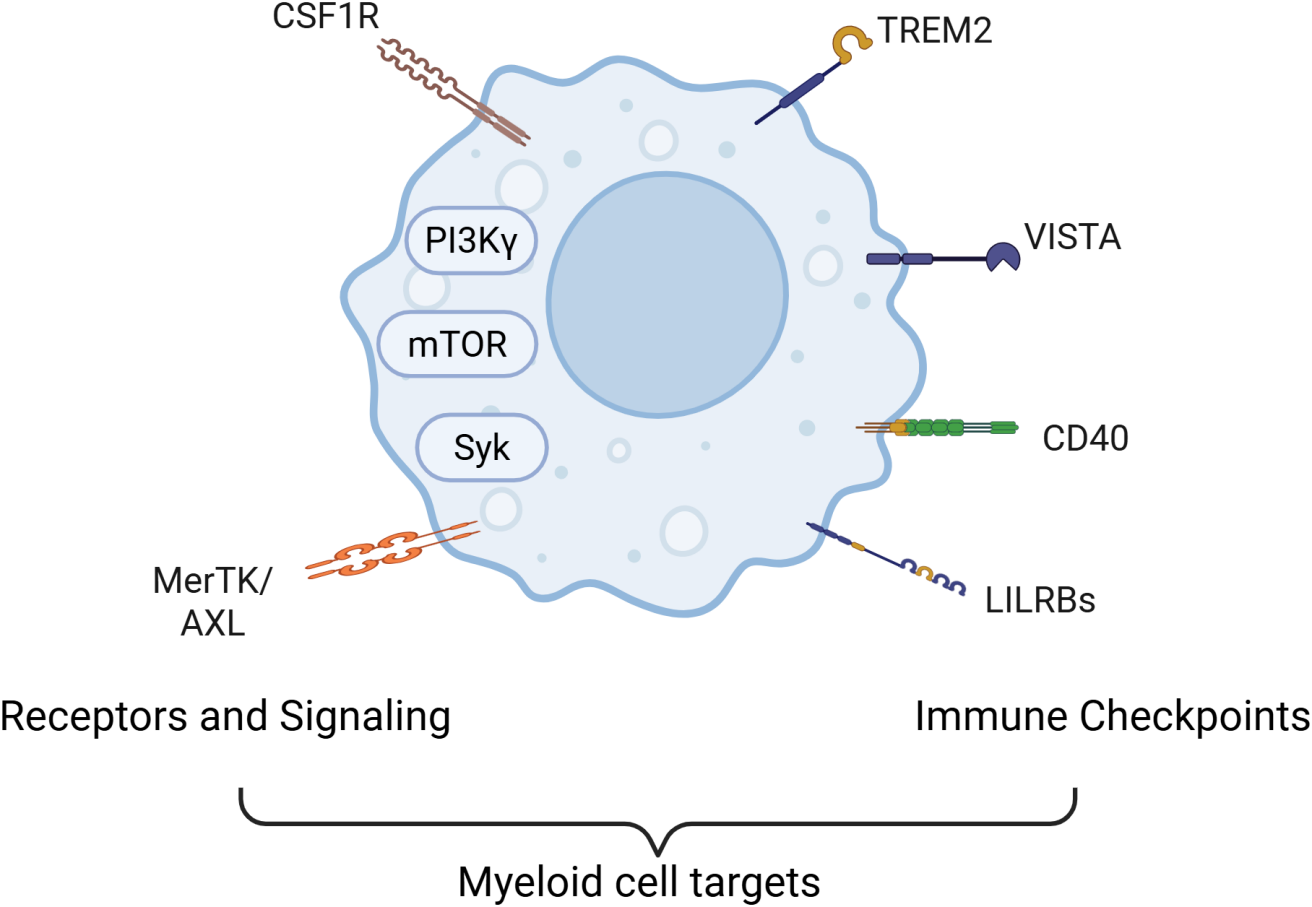
Current strategied for targeting myeloid cells in cancer. Major signaling pathways and cell surface molecules that contribute to the immunosuppressive functions of myeloid cells serve as therapeutic target for altering myeloid cell roles in cancer. Key molecular targets include the PI3Kγ isoform and receptor tyrosine kinases such as CSF1R, which regulate macrophage recruitment, survival, and polarization. Downstream pathways like mTOR and Syk further modulate metabolic and inflammatory programs in tumor-associated macrophages (TAMs) and myeloid-derived suppressor cells (MDSCs). In addition, immunosuppressive signaling through MerTK and Axl receptors contributes to the maintenance of an anti-inflammatory, tumor-supportive myeloid phenotype. Some strategies also target critical immune checkpoint molecules expressed on myeloid cells—including TREM2, LILRB family members, VISTA, and CD40—that regulate antigen presentation, cytokine secretion, and T cell activation. Together, these signaling nodes coordinate a network of immune evasion strategies employed by tumors through myeloid cell modulation.

The purpose of this review is to highlight and critically evaluate recent advances in therapeutic strategies that specifically target myeloid cells within the TME to overcome tumor-induced immunosuppression. In recent years, a growing body of research has focused on designing therapies that either deplete, reprogram, or inhibit the suppressive functions of myeloid cells (8). Agents such as CSF1R inhibitors, CCR2 antagonists, and STAT3 pathway inhibitors have shown promise in preclinical and early-phase clinical studies (9–11). In addition, novel immunotherapies targeting myeloid-specific checkpoints or metabolic regulators are under active investigation. By integrating these strategies into the current immunotherapeutic landscape—either as monotherapies or in combination with checkpoint inhibitors, chemotherapy, or radiation—researchers hope to enhance therapeutic responses, overcome resistance, and achieve more durable outcomes for cancer patients (8). This review highlights key signaling pathways and surface molecules involved in myeloid-mediated immune suppression, including CSF1R, PI3Kγ, mTOR, Syk, MerTK/Axl, and immune checkpoints such as Trem2, LILRBs, VISTA, and CD40 (**Figure 1**). This review also seeks to synthesize these developments and provide insight into the clinical potential, challenges, and future directions of myeloid-targeted cancer immunotherapies. A better understanding of the heterogeneity, plasticity, and context-dependent roles of myeloid cells will be essential for optimizing these treatments and identifying biomarkers for patient selection and response monitoring.

## 2 Myeloid cell biology in tumor immunosuppression

### 2.1 Role of myeloid cells in the tumor microenvironment

**Figure 2:**
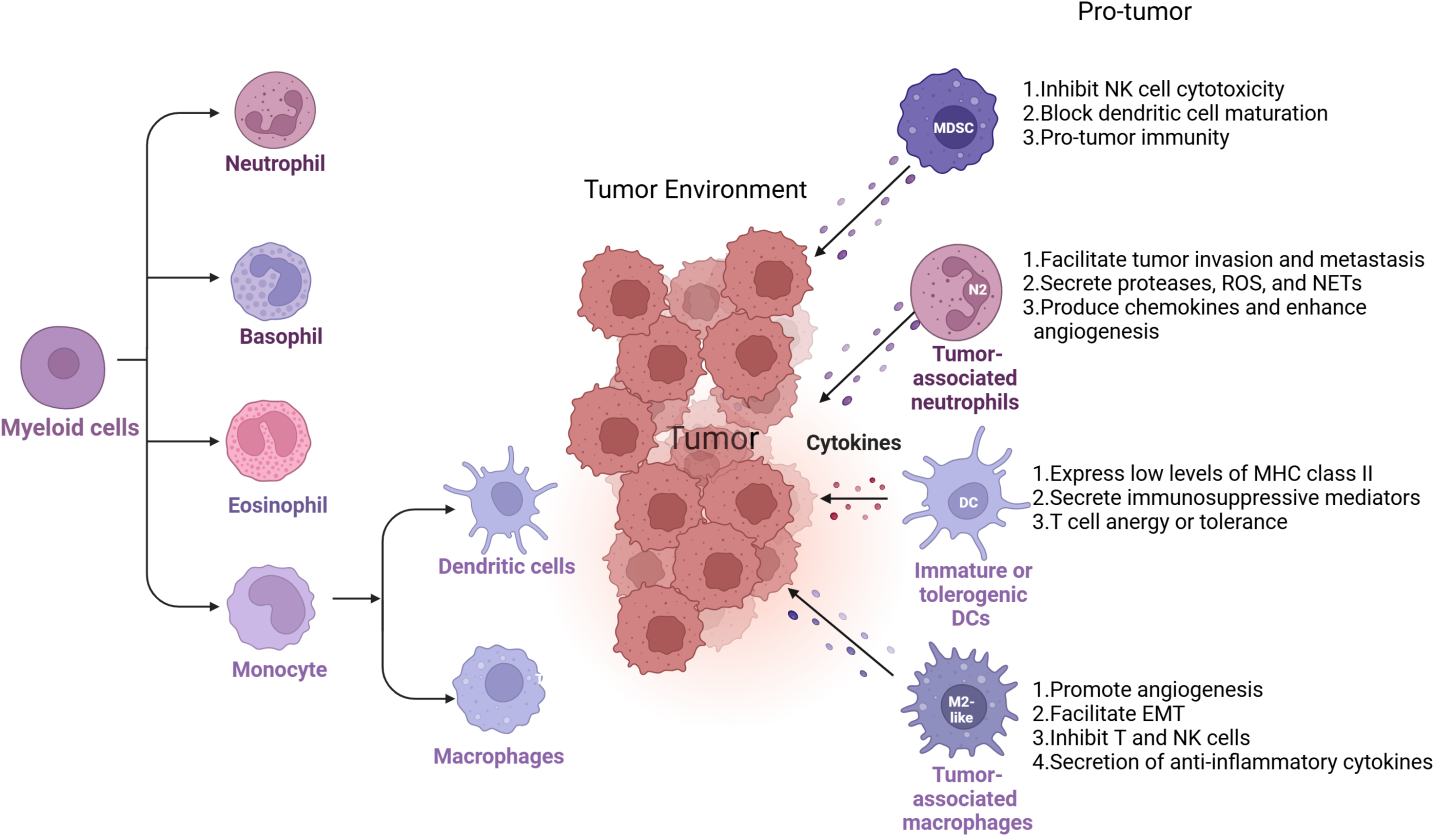
Myeloid cell diversity and plasticity in the TME. Myeloid cells encompass a broad range of immune cell types, including monocytes, eosinophils, basophils, and neutrophils. Within the tumor microenvironment (TME), specific subsets of these cells—such as tumor-associated macrophages (TAMs), myeloid-derived suppressor cells (MDSCs), dendritic cells (DCs), and tumor-associated neutrophils (TANs)—play key roles in promoting tumor progression by orchestrating immunosuppressive networks. Each of these myeloid populations contributes to immune evasion through distinct but complementary mechanisms, including suppression of T cell activation, alteration of cytokine and chemokine profiles, impairment of antigen presentation, and enhancement of angiogenesis and extracellular matrix remodeling. Their functional diversity and plasticity make them important targets for reprogramming strategies in cancer immunotherapy.

Myeloid cells are among the most abundant immune populations in the TME and exhibit remarkable functional plasticity. Derived from hematopoietic progenitors, these cells include monocyte, eosinophil, basophil and Neutrophil (12, **Figure 2**). In the TME, tumor-associated macrophages (TAMs), immune suppressive monocytes and granulocytes (myeloid-derived suppressor cells, MDSCs), dendritic cells (DCs), and tumor-associated neutrophils (TANs) will contribute to tumor immunosuppression (12, **Figure 1**). Within the TME, tumor-derived cytokines and growth factors (e.g., GM-CSF, IL-6, VEGF) drive the recruitment and reprogramming of myeloid cells into pro-tumoral phenotypes (13). This reprogramming shifts their function from immune surveillance and inflammation to immunosuppression, tissue remodeling, angiogenesis, and metastasis facilitation (12).

#### 2.1.1 Tumor-associated macrophages as central orchestrators of the TME

TAMs are often the most abundant immune cells in solid tumors and play a pivotal role in shaping the immunosuppressive landscape of the TME. Initially derived from circulating monocytes and resident macrophages, TAMs are educated by the tumor milieu to adopt an immune suppressive, pro-tumoral phenotype, becoming the population of immune suppressive macrophages (13, 14). Functionally, immunosuppressive macrophages contribute to tumor progression by secreting anti-inflammatory cytokines (IL-10, TGF-β), inhibiting cytotoxic T lymphocyte (CTL) activation, promoting regulatory T cell (Treg) expansion, and supporting angiogenesis through the production of VEGF and matrix metalloproteinases (MMPs) (15, 16). They also remodel the extracellular matrix to create a physical barrier against immune cell infiltration and facilitate metastasis by enhancing tumor cell migration. Through the expression of immune checkpoint ligands such as PD-L1 and VISTA, immunosuppressive macrophages further inhibit T cell function and contribute to immune evasion (17). Their abundance within tumors often corelate with poor prognosis and resistance to therapy (18), underscoring their importance as therapeutic targets in immunomodulation strategies.

#### 2.1.2 MDSCs as potent suppressors of anti-tumor immunity

Myeloid-derived suppressor cells (MDSCs) are immature myeloid cells of the monocyte and granulcyte lineages with strong immunosuppressive activity. In cancer, MDSCs expand significantly and accumulate in the TME under the influence of tumor-secreted factors such as CSF1, G-CSF, IL-6, and prostaglandins (19, 20). These cells suppress both innate and adaptive immunity by multiple mechanisms (21, 22). These cells inhibit T cell and NK cell cytotoxicity and block dendritic cell maturation, creating a layered barrier against effective anti-tumor immunity (17, 23). Their accumulation is associated with tumor burden, metastatic potential, and treatment resistance.

#### 2.1.3 Dendritic cell dysfunction in tumors

Although dendritic cells (DCs) are professional antigen-presenting cells critical for initiating adaptive immunity, their functionality is often impaired in the TME. Tumor-derived cytokines such as IL-10, VEGF, and PGE2 inhibit DC maturation, resulting in accumulation of immature or tolerogenic DCs that fail to effectively prime T cells (24, 25). These dysfunctional DCs often express low levels of MHC class II and co-stimulatory molecules, while secreting immunosuppressive mediators like TGF-β or expressing IDO, contributing to T cell anergy or tolerance rather than activation (26, 27). Consequently, the DC compartment becomes a facilitator rather than a barrier to tumor growth, and restoring DC functionality is a promising avenue for enhancing cancer immunotherapy.

#### 2.1.4 Tumor-associated neutrophils and the pro-tumoral N2 phenotype

Neutrophils are increasingly recognized as influential components of the TME, with tumor-associated neutrophils (TANs) demonstrating dual phenotypes: anti-tumoral (N1) or pro-tumoral (N2). The tumor context, particularly the presence of TGF-β, favors polarization toward the N2 phenotype, which promotes tumor progression through several pathways (28, 29). N2 TANs secrete proteases, ROS, and neutrophil extracellular traps (NETs) that facilitate tumor invasion and metastasis (30). They also produce chemokines such as CXCL1 and CXCL8 that attract additional immunosuppressive cells and enhance angiogenesis (31). Emerging data suggest TANs also regulate pre-metastatic niche formation, positioning them as critical players in both local tumor growth and systemic disease spread.

### 2.2 Mechanisms by which myeloid cells contribute to immunosuppression:

**Figure 3:**
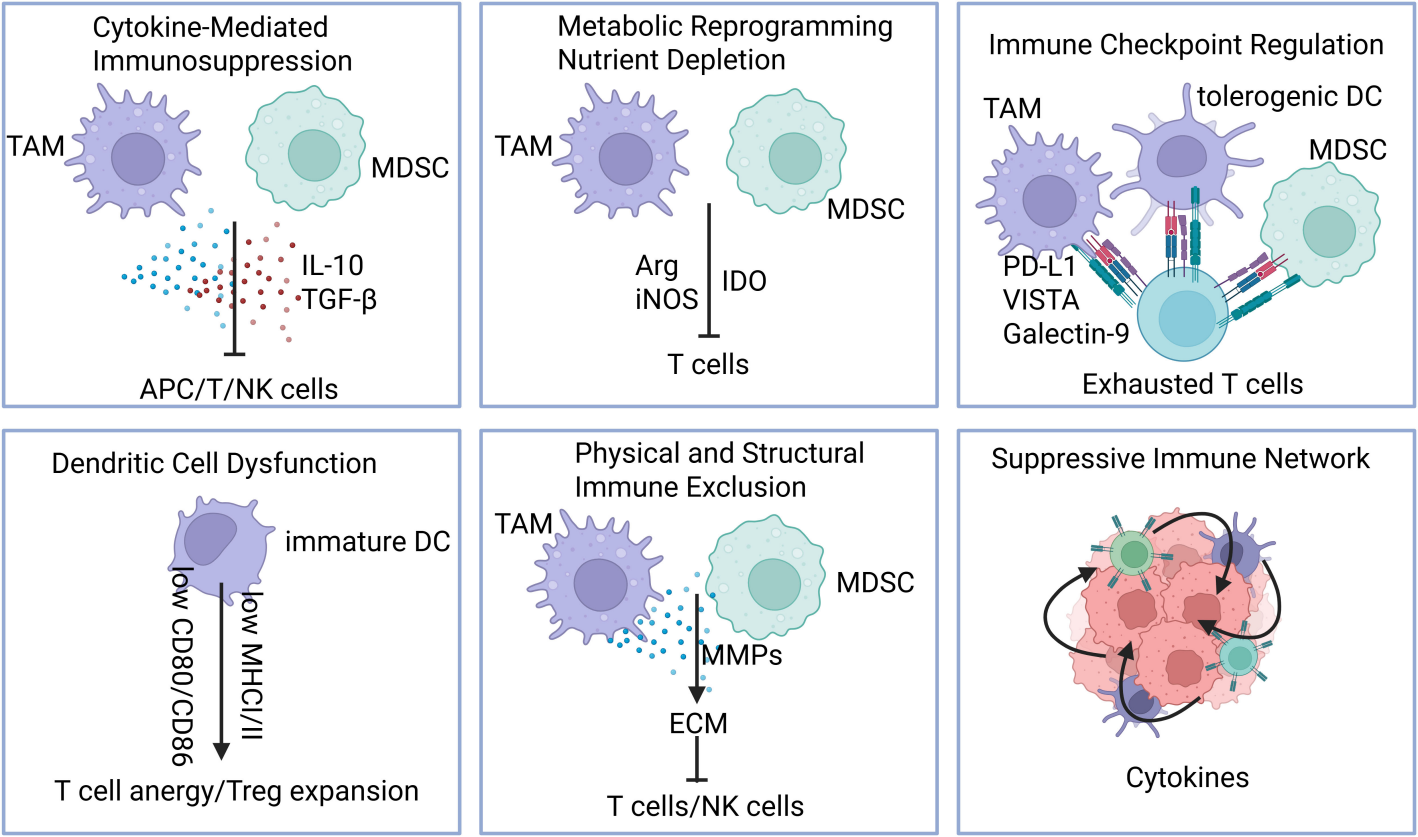
Mechanisms of myeloid cell–mediated immunosuppression. Myeloid cells promote immunosuppression in the tumor microenvironment through multiple, coordinated mechanisms: 1)Cytokine-mediated suppression, including secretion of IL-10, TGF-β, and other anti-inflammatory factors; 2) Metabolic reprogramming and nutrient depletion, such as arginine and production of immunosuppressive metabolites; 3).Immune checkpoint regulation, via expression of PD-L1, VISTA, LILRBs, and other inhibitory ligands; 4) Dendritic cell dysfunction, leading to impaired antigen presentation and T cell priming; 5) Physical and structural immune exclusion, through extracellular matrix remodeling and angiogenesis; 6) Recruitment and stabilization of suppressive networks, including Tregs, MDSCs, and tumor-promoting macrophages.

Myeloid cells contribute to immunosuppression in the tumor microenvironment through several mechanisms as follows (**Figure 3**):

#### 2.2.1 Cytokine-mediated immunosuppression by myeloid cells

One of the primary ways myeloid cells contribute to immunosuppression is through the secretion of anti-inflammatory cytokines, which dampen immune activation and promote tolerance. TAMs and MDSCs secrete high levels of IL-10 TGF-β (17, 32). These cytokines inhibit the function of antigen-presenting cells (APCs), reduce MHC class II expression, and suppress the production of IL-12, which is crucial for Th1 and cytotoxic T lymphocyte responses (33, 34). TGF-β also promotes the expansion of regulatory T cells (Tregs) and blocks the cytotoxic function of CD8^+^ T cells and natural killer (NK) cells, thereby creating an immune-tolerant tumor microenvironment (TME) (34, 35). The cytokine profile skews the immune response away from anti-tumor activity and facilitates tumor progression.

#### 2.2.2 Metabolic reprogramming and nutrient depletion

Myeloid cells suppress T cell function by altering the metabolic landscape of the TME. MDSCs and TAMs express enzymes such as arginase-1 (ARG1), inducible nitric oxide synthase (iNOS), and indoleamine 2,3-dioxygenase (IDO). ARG1 depletes L-arginine, an amino acid essential for T cell proliferation and TCR ζ-chain expression (36–38). Concurrently, iNOS produces nitric oxide (NO), which impairs T cell signal transduction and can induce apoptosis (39). IDO catabolizes tryptophan into kynurenine, a metabolite known to suppress T cell proliferation and promote Treg differentiation (40). Additionally, reactive oxygen species (ROS) generated by myeloid cells cause oxidative stress and nitration of TCRs, leading to functional impairment of tumor-infiltrating lymphocytes (41, 42). These metabolic suppressive pathways represent major barriers to effective antitumor immunity and are under investigation as therapeutic targets.

#### 2.2.3 Immune checkpoint regulation by myeloid cells

Myeloid cells contribute to T cell exhaustion through the expression of immune checkpoint ligands, most notably programmed death-ligand 1 (PD-L1). Inflammatory cytokines such as IFN-γ upregulate PD-L1 on TAMs, MDSCs, and tolerogenic dendritic cells (DCs) (17, 43). PD-L1 binds to PD-1 on activated T cells, leading to reduced proliferation, impaired cytokine secretion, and eventual anergy or apoptosis (44). Beyond PD-L1, myeloid cells also express VISTA (45), Galectin-9 (ligand for TIM-3) (46), and CD155 (ligand for TIGIT) (47), which further inhibit both innate and adaptive immune responses. These checkpoint molecules contribute not only to immune suppression within the TME but also to resistance against immune checkpoint inhibitors, underscoring the importance of targeting myeloid cell-derived checkpoints in combinatorial immunotherapy strategies.

#### 2.2.4 Dendritic cell dysfunction and antigen presentation failure

In the TME, dendritic cells (DCs) often fail to fully mature due to tumor-derived inhibitory signals such as VEGF (48), IL-10 (49), and PGE2 (50). Immature or tolerogenic DCs exhibit low expression of costimulatory molecules (CD80, CD86) and reduced MHC class I/II surface expression, rendering them ineffective at priming naïve T cells (51). Instead of initiating robust cytotoxic responses, these DCs may promote T cell anergy or contribute to Treg expansion. Some DC subsets also express IDO, adding a metabolic suppressive layer to their immunoregulatory role (52). The inability of DCs to function as effective antigen-presenting cells critically impairs the initiation and maintenance of antitumor T cell responses.

#### 2.2.5 Physical and structural immune exclusion

Myeloid cells influence not only cellular but also physical aspects of immunosuppression. TAMs and MDSCs secrete matrix metalloproteinases (MMPs) and other enzymes that remodel the extracellular matrix (ECM), leading to the formation of dense stromal barriers. This ECM restructuring can physically prevent the infiltration of effector T cells and NK cells into the tumor core, a hallmark of “immune-excluded” tumors (53). Additionally, these myeloid populations release vascular endothelial growth factors (VEGF) and other angiogenic factors that promote abnormal vasculature, reducing effective leukocyte trafficking and enhancing tumor perfusion (54). The combination of immune exclusion and vascular dysregulation forms a formidable barrier to immunotherapy penetration.

#### 2.2.6 Recruitment and maintenance of a suppressive immune network

Myeloid cells also play an active role in maintaining a suppressive immune network by secreting chemokines that recruit other regulatory cells. For example, CCL2 recruits monocytes that differentiate into TAMs (14), CXCL12 attracts MDSCs and Tregs (55), and CCL5 enhances the recruitment of Th2 and regulatory populations (56). These feedback loops amplify the immunosuppressive milieu and ensure sustained immune evasion. Moreover, by producing IL-1β and IL-6, myeloid cells can polarize additional macrophages and neutrophils toward tumor-promoting phenotypes, further reinforcing immunosuppression within the TME (57, 58).

## 3 Targeting myeloid cell signaling pathways

### 3.1 Colony-stimulating factor 1 receptor

#### 3.1.1 Role in myeloid cell recruitment and differentiation

CSF1R, a tyrosine kinase receptor expressed primarily on monocytes, macrophages, and other myeloid lineage cells, plays a pivotal role in regulating the recruitment, survival, proliferation, and differentiation of myeloid cells (9, 59). Through paracrine and autocrine signaling, tumor cells and associated stromal elements often produce high levels of CSF1 or IL-34, activating CSF1R on circulating myeloid precursors and promoting their migration into the tumor. Once recruited, CSF1R signaling promotes the differentiation of these precursors into immunosuppressive, M2-like TAMs, which secrete anti-inflammatory cytokines, support tumor angiogenesis and metastasis, and inhibit cytotoxic T cell activity (9, 60).

#### 3.1.2 CSF1R inhibitors and their effects on TAMs and MDSCs

CSF1R inhibitors have emerged as promising immunomodulatory agents in cancer therapy, primarily due to their ability to target TAMs and MDSCs. CSF1R inhibitors, including small-molecule tyrosine kinase inhibitors (e.g., PLX3397, BLZ945) and monoclonal antibodies targeting CSF1R or its ligands, aim to disrupt CSF/IL34-CSF1R signaling axis (9, 59, 61). In preclinical tumor models, CSF1R blockade has been shown to effectively reduce TAM numbers, reprogram TAMs toward a more inflammatory M1-like phenotype, and enhance T cell infiltration and function in glioblastoma, pancreatic ductal adenocarcinoma, breast cancer, lung cancer, melanoma, colon cancer, hepatocellular carcinoma, prostate cancer and sarcoma etc (9, 59–63).

This remodeling of the TME often results in delayed tumor progression and increased sensitivity to immune checkpoint inhibitors such as anti-PD-1/PD-L1 therapies in these cancer types (60–62). However, the response to CSF1R inhibition can vary depending on tumor type, TAM heterogeneity, and compensatory mechanisms (61–63). In preclinical models, CSF1R inhibition reduces the accumulation and immunosuppressive function of MDSCs. Blocking CSF1R signaling impairs MDSC survival and suppresses their ability to produce immunosuppressive cytokines such as IL-10 and TGF-β. This reprogramming of MDSCs leads to enhanced antigen presentation and improved T cell activation, thereby promoting antitumor immunity (62, 63).

#### 3.1.3 Current clinical trials and therapeutic implications

The clinical translation of CSF1R-targeted therapies has gained substantial momentum in recent years, with numerous ongoing and completed clinical trials (**Table 1**) (64–71). Most CSF1R inhibitors under investigation are either small-molecule tyrosine kinase inhibitors (e.g., PLX3397, BLZ945, vimseltinib) or monoclonal antibodies targeting CSF1R (64–71). Pexidartinib, the most clinically advanced CSF1R inhibitor, was approved by the FDA in 2019 for the treatment of tenosynovial giant cell tumor (TGCT) (64).

**Table 1:** Clinical application of CSF1R-targeted therapies in cancer treatment.

Agent Name	Agent Type	Clinical Status/Indication
Pexidartinib (64)	Small molecule	FDA-approved TGCT; trials in solid tumors
BLZ945 (65)	Small molecule	First-in-human oncology trials; terminated glioblastoma combo
DCC-3014 (66)	Small molecule	Approved TGCT; oncology trials ongoing
Emactuzumab (67)	mAb	Phase II TGCT; combos with atezolizumab
AMG 820 (68)	mAb	Phase 1/2 solid tumor trials
Cabiralizumab (69)	mAb	Phase 1 combination trials
IMC-CS4 (70)	mAb	Phase 1 combos with checkpoint inhibitors
Axatilimab (71)	mAb	Oncology and cGvHD trials

However, monotherapy with CSF1R inhibitors has shown limited efficacy in clinical trials across multiple solid tumors. One major limitation is the incomplete depletion or reprogramming of tumor-associated macrophages (TAMs), which often remain functionally immunosuppressive despite treatment (63). Furthermore, tumor heterogeneity and plasticity within the myeloid compartment can lead to compensatory recruitment of other suppressive cells, such as MDSCs or regulatory T cells, reducing the long-term impact of CSF1R blockade (9, 60, 61). In some patients, compensatory upregulation of GM-CSF or IL-34 signaling helps sustain myeloid cell populations despite CSF1R inhibition (63, 66). Clinically, on-target toxicities—such as elevated liver enzymes and cytopenias—can arise due to the disruption of tissue-resident macrophages in healthy organs (63, 68, 70).

These factors, combined with the immune-excluded nature of many tumors, often necessitate the use of CSF1R inhibitors in combination with immune checkpoint inhibitors, chemotherapy, or radiation to achieve meaningful therapeutic responses. As a result, current clinical trials are increasingly focused on combination strategies. These include combining CSF1R inhibitors with immune checkpoint inhibitors, chemotherapy, anti-angiogenic agents, or radiotherapy, aiming to synergistically overcome the immunosuppressive tumor microenvironment (63, 69).

Additionally, biomarkers of response to CSF1R inhibitors are still under development. Despite these challenges, the clinical development of CSF1R inhibitors remains a promising avenue, particularly in combination with other immunotherapies. One of the most studied indicators is the density and phenotype of tumor-associated macrophages (TAMs)—tumors with high infiltration of CD163^+^ or CD206^+^ M2-like TAMs tend to show better responses to CSF1R blockade, as these cells are highly dependent on CSF1R signaling for survival and immunosuppressive function. Elevated intratumoral or circulating levels of CSF1, the ligands for CSF1R, may also predict pathway dependency and therapeutic vulnerability (9, 63). Gene expression profiling of the tumor microenvironment can further identify macrophage-dominant signatures associated with responsiveness. Moreover, post-treatment increases in inflammatory cytokines (e.g., TNF-α, IL-6) or chemokines (e.g., CXCL9, CCL5) may reflect successful TAM reprogramming toward a pro-inflammatory state (9, 60, 61, 63).Despite these advances, no universally validated predictive biomarker exists, and the utility of CSF1, or TAM density varies by tumor type (63). Therefore, integrating multimodal biomarker strategies, such as flow cytometry, IHC, transcriptomics and single cell seq, is increasingly important to stratify patients for CSF1R-targeted therapy.

### 3.2 Phosphoinositide 3-kinase gamma

#### 3.2.1 Role of PI3Kγ in myeloid cell signaling, survival, and migration

PI3Kγ is a class IB isoform of the PI3K family that plays a central role in regulating the function of myeloid cells, particularly monocytes, macrophages, and neutrophils (72, 73). PI3Kγ is predominantly expressed in leukocytes, particularly in myeloid cells, where it is activated downstream of G protein–coupled receptors (GPCRs) and receptor tyrosine kinases in response to chemokines, cytokines, and growth factors (72, 73). Activation of PI3Kγ leads to the production of phosphatidylinositol-3,4,5-trisphosphate (PIP3) at the plasma membrane, initiating a cascade of downstream signaling events that regulateintegrin activation, cytoskeletal rearrangement, chemotaxis, and immune modulation (72–76). PI3Kγ activation promotes myeloid cell recruitment to tumors, where they promote immune evasion and tumor progression (72, 75–77).

#### 3.2.2 Inhibition of PI3Kγ to reduce immunosuppression and enhance anti-tumor immunity

Targeting PI3Kγ has emerged as a promising strategy to reprogram TME and overcome immunosuppression driven by myeloid cells. In tumors, PI3Kγ signaling promotes the accumulation and functional polarization of TAMs and MDSCs toward immunosuppressive phenotypes, contributing to the suppression of cytotoxic T cell activity and facilitating tumor progression (72, 75–78). PI3Kγ inhibition in TAMs led to increased production of pro-inflammatory cytokines, enhanced CD8+ T cell cytotoxicity, and suppressed tumor growth in mouse models of cancers including pancreatic ductal adenocarcinoma, lung adenocarcinoma and colon cancer, attributing to the activation of NF-κB and suppression of C/EBPβ signaling pathways in TAMs (79). Further research by Kaneda and colleagues elucidated the molecular mechanisms underlying this reprogramming. They found that PI3Kγ activity in TAMs stimulates C/EBPβ-dependent expression of immunosuppressive factors like arginase and TGF-β, while suppressing NF-κB-mediated expression of pro-inflammatory cytokines. Inhibition of PI3Kγ reversed these effects, thereby restoring CD8+ T cell-dependent cytotoxicity and inhibiting tumor growth, metastasis and vascular leak (79–82). Collectively, these findings underscore the therapeutic potential of PI3Kγ inhibition in reprogramming TAMs and modulating the TME to promote immune activation and enhance anti-tumor immunity.

#### 3.2.3 Therapeutic potential and challenges in targeting PI3Kγ clinically

The clinical targeting of PI3Kγ has emerged as a promising approach to overcome myeloid-mediated immunosuppression and improve cancer immunotherapy outcomes (83–86). Eganelisib (IPI-549), a selective PI3Kγ inhibitor, has shown encouraging early results across multiple clinical settings (83–86). In the MARIO-1 Phase 1/1b trial, eganelisib demonstrated manageable toxicity and preliminary signs of clinical benefit when administered either as monotherapy or in combination with nivolumab in patients with advanced solid tumors (83, 84). Subsequent Phase II analyses in patients with advanced urothelial carcinoma (MARIO-275) indicated that eganelisib combined with nivolumab led to numerically higher objective response rates compared to nivolumab alone, suggesting enhanced immunotherapeutic synergy through modulation of the tumor microenvironment (85). Moreover, a recent study in metastatic triple-negative breast cancer (mTNBC) showed that combining eganelisib with checkpoint inhibitors and chemotherapy resulted in macrophage reprogramming toward a pro-inflammatory phenotype, increased CD8^+^ T cell infiltration, and extracellular matrix remodeling, supporting the mechanism of action observed in preclinical models (86). In summary, continued investigation in clinical trials, coupled with mechanistic insights into biomarker development, will be essential for fully realizing the clinical potential of PI3Kγ inhibitors and integrating them into next-generation immunotherapy regimens (87).

PI3Kγ inhibitors have shown strong immunomodulatory effects in preclinical models. Their translation into clinical success, like all that of other myeloid cell inhibitors, has necessitated combination with T cell targeting and/or tumor cell targeting therapies. The selection of the best combination therapies to pair with myeloid targeted theapies for cancer clinical trials can be challenging, as a diverse array of therapies can be applied to each cancer type, and multiple trials may be required to ensure clinical success. Furthermore, although predictive biomarkers such as low MHCII levels on circulating CD14+ myeloid cells and tumor PD-L1 status were evaluated in patients treated with eganelisib, patients with and without these biomarkers showed clinical responses. Currently, no definitive biomarker identifies those patients most likely to benefit from PI3Kγ-targeted therapy (83–86). Finally, PI3Kγ inhibitors, most myeloid targeted therapeutics have resulted in serum elevations in two key liver enzymes, AST and ALT, although these elevations have been transient and reversible.Together, these challenges underscore the need for continued investigation of combination approaches, refined dosing regimens, and biomarker-guided clinical development to fully realize the potential of PI3Kγ inhibitors and other myeloid targeting therapeutics in immuno-oncology.

### 3.3 Mechanistic target of rapamycin

#### 3.3.1 mTOR signaling in myeloid cells and its role in the TME

mTOR is a central regulator of cellular metabolism, growth, and immune function, and plays a pivotal role in shaping the phenotype and function of myeloid cells in the TME (88, 89). mTOR functions through two distinct complexes: mTORC1 and mTORC2, which are activated downstream of growth factors, nutrients, and immune stimuli. In myeloid cells, mTOR signaling integrates metabolic and inflammatory cues to direct cell fate decisions, cytokine production, and immunosuppressive activity (88, 89). In macrophages, mTORC1 activation is generally associated with immune suppressive, anti-inflammatory polarization, contributing to immune suppression, angiogenesis, and tumor progression (79, 90). Similarly, in MDSCs, mTOR activity supports expansion and suppressive function by regulating glucose metabolism and promoting expression of immunosuppressive mediators (91). mTOR also governs dendritic cell maturation and function, where aberrant mTOR signaling can result in the accumulation of immature or tolerogenic DCs that fail to stimulate effective T cell responses, further weakening antitumor immunity (92).

#### 3.3.2 Impact of mTOR inhibitors on TAMs and MDSCs and synergistic effects with other immunotherapies

The mTOR signaling pathway has been extensively investigated for its role in signaling downstream of tumor cell oncogenes such as PIK3CA and KRAS. Inhibitors of mTOR were developed as inhibitors of tumor cell proliferation and survival. However, the inhibition of the mTOR signaling pathway has emerged as a promising strategy to modulate the function of immunosuppressive myeloid cells in the TME. mTOR inhibitors, including rapamycin (sirolimus) and its analogs (everolimus, temsirolimus), can disrupt these processes and reprogram the immunosuppressive function of TAMs and MDSCs (93, 94). In preclinical tumor models, mTOR inhibition has been shown to reduce the accumulation of MDSCs in tumors and to promote the differentiation of TAMs into M1-like, inflammatory macrophages capable of enhancing antigen presentation and stimulating T cell activation in hepatocellular carcinoma, breast cancer, lung cancer, colorectal cancer, melanoma, ovarian cancer and pancreatic cancer (93, 94). Importantly, mTOR inhibitors have demonstrated synergistic potential when combined with immune checkpoint blockade therapies by reducing TAM and MDSC-mediated suppression (94). However, the use of mTOR inhibitors requires careful dosing and timing, as mTOR is also critical for the proliferation and function of effector T cells (95).

#### 3.3.3 Clinical status for mTOR inhibitors

The clinical development of mTOR inhibitors for cancer therapy has significantly advanced over the past two decades (96–105). The most established mTOR inhibitors are rapamycin (sirolimus) and its analogs, which include everolimus, temsirolimus, and ridaforolimus (96–100). The clinical benefits of first-generation mTOR inhibitors have been limited by modest overall response rates, cytostatic rather than cytotoxic activity, and the development of adaptive resistance mechanisms. To address this, second-generation mTOR inhibitors—such as dual mTORC1/mTORC2 inhibitors (e.g., sapanisertib, vistusertib) and dual PI3K/mTOR inhibitors (e.g., dactolisib, apitolisib)—have been developed and are undergoing evaluation in early-phase clinical trials (101–105).

While preclinical data suggest these therapeutics also alter myeloid cell properties in tumors, it is unclear whether these drugs affected myeloid cells in these clinical studies. Although these mTOR inhibitors have shown clinical efficacy in select cancers, their broader application as myeloid compartment therapeutics is limited. Feedback activation of upstream pathways, especially AKT and PI3K, due to mTORC1 inhibition, can undermine therapeutic efficacy and promote resistance (106). mTOR inhibitors can also dampen antitumor T cell responses and thus complicate their use in combination with immunotherapies (106, 107). Toxicities such as stomatitis, hyperlipidemia, hyperglycemia, and non-infectious pneumonitis limit dose intensity and long-term administration in some patients (96–102). The clinical benefits of mTOR inhibitors have been limited by modest overall response rates, and the development of adaptive resistance mechanisms (106–108). It is possible that these drugs target myeloid cells as well as tumor cells and could be used in limited doses in combination with checkpoint inhibitors and other therapeutics.Preclinical studies have demonstrated that transient or combinatorial mTOR inhibition can enhance the efficacy of immune checkpoint blockade therapies, and several combination regimens are being evaluated clinically96,97.

Predictive biomarkers for mTOR inhibitor response remain an area of active investigation, given the variable efficacy of these agents across different tumor types. Recent studies have also suggested that baseline immune context—levels of myeloid-derived suppressor cells (MDSCs) or T cell exhaustion markers—may influence responsiveness, especially when mTOR inhibitors are combined with immunotherapy (88, 92, 93). However, no single biomarker has been validated across cancer types, and heterogeneity within the tumor and the tumor microenvironment may limit the predictive power of biomarker of response. Consequently, a composite biomarker strategy incorporating genomic, proteomic, and immune parameters may be required to accurately identify patients who are most likely to benefit from mTOR-targeted therapy (106–108). Ongoing studies are focused on identifying further biomarkers of response, refining patient selection, and determining optimal dosing strategies that balance anti-tumor efficacy with preservation of immune function (108).

### 3.4 Spleen tyrosine kinase

#### 3.4.1 Function of Syk in myeloid cell activation and immune response modulation

Syk is a non-receptor tyrosine kinase that plays a pivotal role in myeloid cell activation, functioning as a central mediator in immune receptor signaling (109, 110). Expressed in various innate immune cells, Syk is primarily activated downstream of immunoreceptors (110). Upon ligand engagement, Syk is recruited to phosphorylated ITAM motifs, where it initiates intracellular signaling cascades that regulate cytokine production, phagocytosis, oxidative burst, cellular adhesion, and antigen presentation (109–111). In macrophages and dendritic cells, Syk activation contributes to the induction of pro-inflammatory cytokines, which are critical for orchestrating innate immune responses and bridging to adaptive immunity (100, 101). Syk also modulates antigen processing and presentation (109, 110).

#### 3.4.2 Therapeutic inhibition of Syk in reprogramming the TME

Therapeutic targeting of Syk has emerged as a novel strategy for reprogramming the TME by modulating the immunosuppressive functions of myeloid cells, particularly TAMs and MDSCs (111–113). In TAMs, Syk activation supports an M2-like, immunosuppressive profile, suppression of T cell activity, and promotion of angiogenesis and extracellular matrix remodeling (113). Syk also facilitates the crosstalk between TAMs and other suppressive cells, including Tregs and MDSCs, contributing to an immune-excluded or immune-silent TME in breast cancer, pancreatic ductal adenocarcinoma, colorectal cancer, ovarian cancer, lung cancer and melanoma (111–114).

Inhibition of Syk has been shown to disrupt these signaling circuits. For example, in models of chronic lymphocytic leukemia (CLL), the Syk inhibitor fostamatinib (R788) reduced tumor cell survival and disrupted B cell receptor (BCR) signaling, which is crucial for leukemic cell maintenance (115, 116). Syk inhibition also impaired the expansion and suppressive activity of myeloid-derived suppressor cells (MDSCs) in certain settings, suggesting broad potential for modulating the TME (112–115).

#### 3.4.3 Clinical findings for Syk inhibitors

Clinically, several Syk inhibtors had been in clinical trials (**Table 2**) (115–120). Fostamatinib, an oral Syk inhibitor developed for immune thrombocytopenia, has been repurposed for oncology. In early-phase clinical trials involving patients with hematologic malignancies, including CLL and diffuse large B-cell lymphoma (DLBCL), fostamatinib showed evidence of disease stabilization and partial responses, particularly in heavily pretreated patients (115). Another Syk inhibitor, entospletinib (GS-9973), was developed as a second-generation agent with improved selectivity and pharmacokinetics (116, 117). In the solid tumor setting, the application of Syk inhibitors is still in the exploratory phase (116–120).

**Table 2:** Advances in the clinical development of Syk-Selective inhibitors for cancer therapy.

Agent Name	Agent Selectivity	Clinical Stage/Indications
Fostamatinib (115)	Syk-selective	Approved for ITP; Phase I in ovarian and solid tumors
Entospletinib (116, 117)	Syk-selective	Phase I/II in CLL, DLBCL, MCL, NHL, AML, GvHD
Cerdulatinib (118)	Syk/JAK dual	Phase I/II in T-cell and B-cell lymphomas
Sovleplenib (119)	Syk-selective	Phase II for autoimmune hematologic disease; expansion planned to hematologic cancers
TAK-659 (120)	Syk/FLT3 dual	Phase I in advanced B-cell lymphomas

Despite promising preclinical data, the clinical development of Syk inhibitors has been limited by several challenges. One major issue is the pleiotropic role of Syk in different cell types, which will raise concerns about off-target immunosuppression or unexpected immune dysregulation when systemically inhibited (121). Additionally, single-agent efficacy of Syk inhibitors in solid tumors has been modest, possibly due to the redundancy of downstream pathways and compensatory signaling (114, 115). Pharmacokinetically, early Syk inhibitors such as fostamatinib have shown limited selectivity and off-target effects, leading to toxicity profiles including cytopenias, hypertension, and gastrointestinal adverse events (115). Finally, the lack of validated biomarkers for patient stratification or pharmacodynamic monitoring makes it difficult to identify responsive patients and optimize treatment regimens in clinical trials. Ongoing research is focused on identifying predictive biomarkers, optimizing dosing regimens, and developing more selective Syk inhibitors to maximize efficacy while minimizing systemic toxicity in clinical trails (121).

### 3.5 MerTK/Axl (tyrosine kinase receptors)

#### 3.5.1 Role of MerTK and Axl in myeloid cell polarization and immune tolerance

MerTK and Axl are receptor tyrosine kinases that play critical roles in the regulation of innate immunity, particularly in the polarization of myeloid cells such as macrophages, DCs, and MDSCs. These receptors are activated by ligands such as GAS6 and Protein S, often in complex with phosphatidylserine (PtdSer) exposed on apoptotic cells (122). Upon ligand engagement, MerTK and Axl signaling cascades that promote anti-inflammatory responses, phagocytosis of apoptotic cells (efferocytosis), and the maintenance of immune tolerance (123). In the TME, MerTK and Axl are frequently upregulated in TAMs, where they promote an M2-like immunosuppressive phenotype. This polarization is leading to suppression of CD8^+^ T cell activity and facilitation of tumor growth, angiogenesis, and tissue remodeling (122, 123). Additionally, Axl signaling in dendritic cells inhibits maturation and antigen presentation, further weakening adaptive immune responses (124, 125). As such, MerTK and Axl are central to establishing and sustaining a tolerogenic, tumor-permissive environment.

#### 3.5.2 Inhibition strategies targeting MerTK/Axl to overcome myeloid-driven suppression

MerTK and Axl have become promising targets for cancer immunotherapy, particularly in tumors with a high burden of myeloid-driven immune suppression. Several inhibition strategies have been developed, including small-molecule tyrosine kinase inhibitors (TKIs), monoclonal antibodies, and decoy receptors that interfere with ligand binding.Selective TKIs such as bemcentinib (BGB324) for Axl (126) and MRX-2843 for MerTK/FLT3 dual inhibition (127) have shown potential in blocking immune suppressive macrophage polarization, restoring antigen presentation, and enhancing pro-inflammatory cytokine production. These effects lead to increased T cell infiltration and anti-tumor immune responses (126–128).

In preclinical studies, bemcentinib (BGB324), a selective Axl inhibitor, has demonstrated the ability to block immunosuppressive macrophage polarization and restore antigen presentation in models of non-small cell lung cancer (NSCLC), triple-negative breast cancer (TNBC), melanoma, and pancreatic ductal adenocarcinoma (PDAC). These effects led to enhanced pro-inflammatory cytokine production and T cell infiltration, contributing to more effective anti-tumor immune responses (126). Similarly, MRX-2843, a dual MerTK/Axl inhibitor, has shown immune-modulatory activity in acute myeloid leukemia (AML), lung adenocarcinoma, ovarian cancer, and pediatric gliomas, where it reprogrammed tumor-associated macrophages and improved T cell function (127). These findings highlight the therapeutic potential of TAM receptor blockade across both solid tumors and hematologic malignancies.

Importantly, MerTK/Axl inhibition also reduces the accumulation and suppressive function of MDSCs, further relieving immune suppression (128). Combination therapies are actively being explored, pairing MerTK/Axl inhibitors with checkpoint inhibitors, chemotherapy, or radiation therapy to synergistically boost immune responses and overcome resistance to monotherapies (125, 129, 130). For example, in pancreatic cancer and breast cancer models, combining Axl inhibition (e.g., bemcentinib) with radiation therapy or chemotherapy (such as paclitaxel or gemcitabine) reprograms tumor-associated macrophages (TAMs), increases antigen presentation, and improves CD8^+^ T cell infiltration, leading to more robust immune responses (125). In non-small cell lung cancer (NSCLC) and melanoma, Axl inhibitors have been paired with checkpoint blockade therapies (e.g., anti–PD-1 or anti–PD-L1), which can convert immune-excluded tumors into inflamed, T cell–infiltrated environments (125). Similarly, MRX-2843, a dual MerTK/Axl inhibitor, has shown preclinical synergy with checkpoint inhibitors in AML, gliomas, and ovarian cancer by blocking myeloid-driven immune suppression (130). These combination strategies aim to neutralize immunosuppressive cues from the tumor microenvironment and restore effective anti-tumor immunity that is often limited with monotherapy approaches.

#### 3.5.3 Evidence from human studies

In patient studies, elevated expression of MerTK and Axl in TAMs or circulating monocytes has been correlated with poor prognosis, advanced disease stage, and resistance to immunotherapy in cancers (131–135). Clinical trials of bemcentinib (BGB324), a selective Axl tyrosine kinase inhibitor, have demonstrated promising activity in a range of Axl-expressing cancers, both as monotherapy and in combination with immune checkpoint inhibitors. In a Phase II trial (NCT03184571), bemcentinib combined with pembrolizumab showed signs of clinical benefit in Axl-high non-small cell lung cancer (NSCLC), particularly in patients with limited response to prior therapies. Similarly, in mesothelioma, the combination of bemcentinib and durvalumab (anti–PD-L1) improved immune infiltration and disease stabilization in patients who had previously progressed on standard treatment. Monotherapy studies have also shown that bemcentinib is well tolerated and has disease-modulating effects in acute myeloid leukemia (AML) and triple-negative breast cancer (TNBC), especially in tumors exhibiting epithelial–mesenchymal transition (EMT) phenotypes driven by Axl. These studies support the rationale for targeting Axl to sensitize tumors to immunotherapy and overcome resistance mechanisms associated with immune exclusion and myeloid-derived suppression (131–134). MRX-2843 is a first-in-class, orally available small-molecule dual inhibitor of MerTK and FLT3 that is currently under clinical investigation for both hematologic malignancies and solid tumors. In a Phase I clinical trial (NCT04848116), MRX-2843 has shown a favorable safety profile and early signs of anti-tumor activity in patients with relapsed or refractory acute myeloid leukemia (AML) and T-cell acute lymphoblastic leukemia (T-ALL), where aberrant MerTK and FLT3 signaling drive disease progression and therapy resistance. Additionally, MRX-2843 has been granted Orphan Drug Designation for AML and T-ALL by the FDA. The agent is also being explored in early-phase trials for pediatric gliomas, where MerTK is implicated in immune evasion and tumor proliferation. In these studies, MRX-2843 not only directly targets leukemic cells but also reprograms the tumor microenvironment by suppressing immunosuppressive macrophage signaling, offering a dual mechanism of action through both tumor-intrinsic and immune-mediated pathways (127, 128, 130, 135).Together, these findings underscore the importance of MerTK and Axl in myeloid-mediated immune tolerance and provide a strong rationale for their continued development as therapeutic targets to reprogram the TME and improve cancer immunotherapy outcomes.

Despite compelling preclinical evidence showing that MerTK and Axl inhibitors can suppress immunosuppressive macrophage polarization, enhance antigen presentation, and improve response to checkpoint inhibitors, their clinical development faces multiple challenges. One major issue is the broad expression of TAM receptors (Tyro3, Axl, MerTK) across both tumor and normal tissues, which increases the risk of off-tumor effects such as impaired clearance of apoptotic cells and autoimmune-like toxicities. Another challenge is functional redundancy among TAM receptors. Inhibiting Axl or MerTK alone may be insufficient due to compensatory upregulation of other family members, potentially limiting therapeutic efficacy (136–138). Clinically, limited biomarker availability hinders patient stratification. While Axl expression correlates with poor prognosis in many cancers, predictive biomarkers for inhibitor sensitivity remain undefined. Additionally, toxicity profiles, including thrombocytopenia, hepatic enzyme elevation, and fatigue, have been reported in early-phase trials, necessitating dose reductions that may compromise efficacy (132–134). Finally, the heterogeneity of the tumor microenvironment (TME) may influence response to MerTK/Axl inhibition, emphasizing the need for rational combination strategies and robust pharmacodynamic markers in future clinical development (136–138).

## 4 Immune checkpoints in myeloid cells

### 4.1 Triggering receptor expressed on myeloid cells 2

#### 4.1.1 Roles for TREM2 in macrophage function and tumor progression

Triggering Receptor Expressed on Myeloid Cells 2 (TREM2) is a transmembrane receptor predominantly expressed on myeloid cells, including macrophages, dendritic cells, and microglia that recognizes phospholipids and triggers phagocytosis of apoptotic cells (139). In the TME, unlike classical pattern recognition receptors (e.g., TLRs, CSF1R, CD40) that primarily trigger immune activation, inflammation, or differentiation, TREM2 mainly promotes cell survival, lipid metabolism, and anti-inflammatory tissue-remodeling functions. TREM2 is highly expressed on a subset of TAMs that exhibit immunosuppressive, tissue-remodeling, and pro-tumorigenic properties (140). Unlike other receptors, upon engagement with endogenous ligands—such as phospholipids, apoptotic cells, and lipoproteins—TREM2 signals through the adaptor protein DAP12, leading to phosphorylation of its ITAM motif and activation of downstream pathways including PI3K-AKT and ERK, thereby promoting cell survival, proliferation, and anti-inflammatory cytokine production (139–141). In the tumor microenvironment (TME), TREM2+ macrophages display a transcriptional program enriched for immunosuppressive mediators such as IL-10, TGF-β, and arginase-1 (ARG1), and show reduced expression of antigen-presentation molecules (e.g., MHC-II) (142, 143). Functionally, these TREM2+ tumor-associated macrophages (TAMs) inhibit cytotoxic T lymphocyte (CTL) recruitment and activation, facilitate extracellular matrix (ECM) remodeling, and promote an immune-excluded tumor architecture. Moreover, TREM2 signaling facilitates immune evasion by creating a suppressive niche that limits dendritic cell maturation and T cell recruitment (143–145). TREM2 signaling also suppresses the production of inflammatory cytokines like IL-12 and TNF-α, which are critical for priming effective T cell responses (140, 141). In summary, TREM2^+^ TAMs are immunosuppressive, and TREM2 blockade restores anti-tumor immunity (139).

#### 4.1.2 Strategies to inhibit TREM2 signaling to enhance anti-tumor immunity

**Figure 4:**
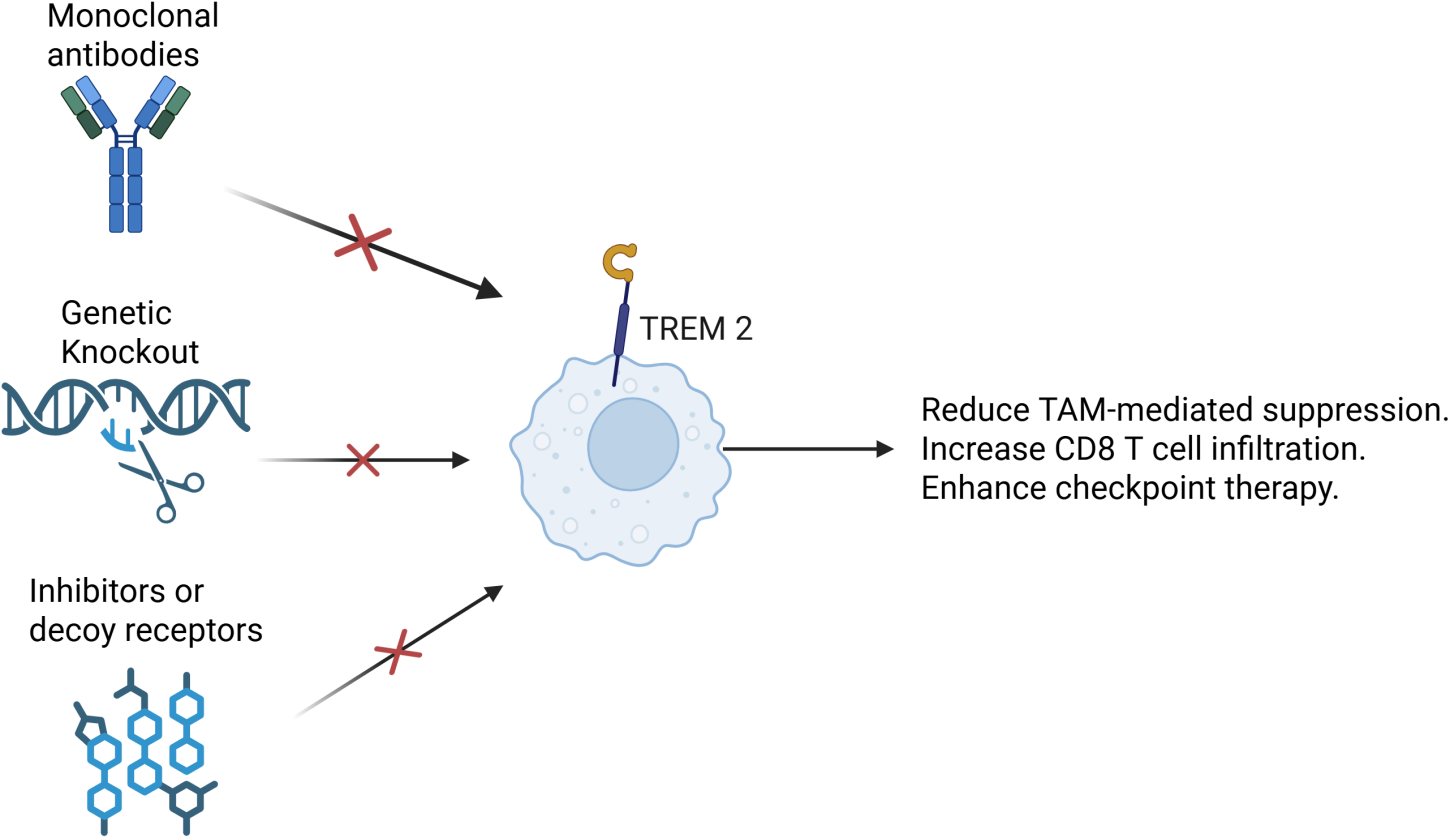
Targeting TREM2. Several therapeutic strategies have been developed to target TREM2. One approach involves the use of monoclonal antibodies that either block the interaction between TREM2 and its ligands or mediate antibody-dependent cellular cytotoxicity (ADCC), effectively depleting TREM2^+^ TAMs. Another strategy relies on genetic methods such as CRISPR-Cas9-mediated knockout or conditional ablation of TREM2 in preclinical models. These tools allow researchers to investigate how the loss of TREM2 affects macrophage phenotype, cytokine production, and overall anti-tumor immunity. Additionally, small-molecule inhibitors and engineered decoy receptors have been designed to interfere with TREM2-DAP12 signaling, a key pathway that transmits immunosuppressive signals within TAMs. Collectively, these approaches aim to reprogram TAMs toward a more pro-inflammatory state, enhance CD8^+^ T cell infiltration and activity, and improve the efficacy of immune checkpoint blockade. By alleviating the suppressive influence of myeloid cells, TREM2-targeted therapies offer a promising avenue for overcoming resistance to immunotherapy in a range of solid tumors.

Targeting TREM2 presents a novel immunotherapeutic strategy aimed at reprogramming the myeloid compartment and enhancing anti-tumor immune responses. Inhibiting TREM2 signaling can disrupt the immunosuppressive functions of TREM2^+^ TAMs and convert them into a more pro-inflammatory, antigen-presenting phenotype (139). Several approaches have been developed to achieve this, including (**Figure 4**): 1). Monoclonal antibodies that block TREM2 ligand binding or induce antibody-dependent depletion of TREM2^+^ TAMs (145); 2). Genetic ablation or CRISPR-mediated knockout of TREM2 in preclinical models to assess the effects on macrophage function and tumor immunity (146); 3). Small-molecule inhibitors or decoy receptors to prevent TREM2-DAP12 signaling activation (145, 146). These strategies aim to reduce TAM-mediated suppression, increase CD8^+^ T cell infiltration, and improve the efficacy of checkpoint inhibitors.Importantly, combination strategies—such as TREM2 blockade plus anti–PD-1/PD-L1 therapy—are being actively explored due to their synergistic effects in reversing T cell exclusion and enhancing tumor clearance (147–149).

#### 4.1.3 Preclinical studies and early-phase clinical trials for targeting Trem2

Preclinical studies across various cancer types suggest that TREM2 is a promising therapeutic target for cancer immunotherapy.In non-small cell lung cancer (NSCLC), TREM2^+^ TAMs are enriched in immunologically “cold” tumors and correlate with poor response to anti–PD-1 therapy. Inhibiting TREM2 signaling reprograms these cells toward a pro-inflammatory phenotype, increasing MHC-II and co-stimulatory molecule expression, which restores CD8^+^ T cell infiltration and improves responsiveness to immune checkpoint blockade (140, 142). In hepatocellular carcinoma (HCC), TREM2 is highly expressed on myeloid cells in the tumor microenvironment and drives an immunosuppressive program. Genetic deletion or antibody-mediated blockade of TREM2 in HCC models leads to enhanced IL-12 production and antigen presentation capacity, facilitating stronger T cell activation and reduced tumor growth (145, 147). In colorectal cancer (CRC), TREM2^+^ TAMs contribute to immune evasion through suppression of dendritic cell recruitment and cytokine production. TREM2 knockout in murine CRC models results in a shift toward an M1-like TAM phenotype, marked by increased TNF-α and IL-1β, and restores local cytotoxic T cell activity (146). In pancreatic ductal adenocarcinoma (PDAC), a tumor type typically resistant to immunotherapy, TREM2 expression defines a distinct subset of TAMs that limit antigen presentation and T cell entry. Blocking TREM2 signaling promotes cross-presentation of tumor antigens, boosts CD80/CD86 levels, and synergizes with anti–PD-1 therapy to control tumor growth (142, 143). In triple-negative breast cancer (TNBC) and other aggressive breast cancers, TREM2^+^ macrophages accumulate within the tumor stroma and suppress effective immune priming. Antibody-mediated TREM2 inhibition reprograms these macrophages into a pro-inflammatory, T cell–recruiting phenotype, enhancing therapeutic responses to immunotherapy. In melanoma, single-cell transcriptomic analyses reveal that TREM2 is upregulated on immunosuppressive TAMs associated with immune checkpoint resistance. TREM2 blockade in murine melanoma models results in enhanced IFN-γ signaling, macrophage repolarization, and a more permissive environment for T cell–mediated tumor clearance. In glioblastoma, where the microenvironment is heavily myeloid-dominated, TREM2 is expressed on both TAMs and microglia, promoting tumor-supportive inflammation. Genetic deletion of TREM2 delays glioma progression, partly through increased antigen presentation and recruitment of cytotoxic lymphocytes (150–152).

Encouraged by these findings, early-phase clinical trials are underway to evaluate TREM2-targeted agents in humans. One of the leading therapeutic candidates is PY314, a humanized anti-TREM2 monoclonal antibody developed by Pionyr Immunotherapeutics, which is currently being evaluated in a Phase I clinical trial (NCT04691375) in patients with advanced solid tumors, including non-small cell lung cancer (NSCLC), renal cell carcinoma, and colorectal cancer. This trial is evaluating the safety, pharmacokinetics, and preliminary efficacy of PY314 both as monotherapy and in combination with nivolumab, an anti–PD-1 checkpoint inhibitor (153–155).

Although TREM2 inhibition has emerged as a promising strategy, its clinical application faces several key challenges. TREM2^+^ macrophages are often enriched in immune-excluded or “cold” tumors, but their distribution and functional role vary greatly across cancer types and patient populations, complicating patient selection and trial design clinically. Furthermore, TREM2 plays a dual role in tissue homeostasis and inflammation. While blocking TREM2 may enhance antigen presentation and T cell infiltration, it also carries the risk of overactivating myeloid cells, potentially leading to uncontrolled inflammation or autoimmune-like adverse events, particularly in organs with a high density of tissue-resident macrophages. From a pharmacological standpoint, the development of TREM2-targeted agents is still in early clinical stages. Most data are preclinical, and human safety and efficacy profiles remain largely undefined. Additionally, TREM2 lacks robust circulating biomarkers, making it difficult to monitor target engagement or stratify responders in ongoing trials. In some models, TREM2^+^ macrophages may coexist with other immunosuppressive populations, suggesting that TREM2 inhibition alone may not fully remodel the tumor microenvironment, thus requiring combination therapies. Lastly, heterogeneity in TREM2 gene expression and mutations raises the possibility of off-tumor risks or long-term effects on central immune regulation, necessitating careful patient screening and extended follow-up in clinical settings (143, 145, 150, 153, 155). Future directions for TREM2 inhibition include identifying predictive biomarkers for response, optimizing combination regimens, and expanding indications to other immunologically resistant cancers (155).

### 4.2 Leukocyte immunoglobulin-like receptor B

#### 4.2.1 Role of LILRBs in myeloid cell inhibition and tumor immune evasion

The Leukocyte Immunoglobulin-like Receptor Subfamily B (LILRB) family comprises inhibitory receptors predominantly expressed on myeloid cells such as macrophages, monocytes, dendritic cells, and certain subsets of granulocytes (156). Members of this family, including LILRB1–LILRB5, contain immunoreceptor tyrosine-based inhibitory motifs (ITIMs) in their cytoplasmic domains, which, upon ligand binding, recruit phosphatases such as SHP-1 and SHP-2 to suppress activating signaling pathways (156, 157). In the TME, LILRBs are often co-opted to maintain an immunosuppressive myeloid phenotype, particularly in TAMs (158) and MDSCs (159). Engagement of LILRBs by ligands such as MHC class I molecules, leads to the inhibition of myeloid activation, decreased pro-inflammatory cytokine secretion, and impaired antigen presentation (159, 160).

In non-small cell lung cancer (NSCLC), LILRB2 is highly expressed on tumor-associated macrophages (TAMs) and dendritic cells, contributing to immune suppression and poor antigen presentation. Preclinical models show that LILRB2 blockade reprograms myeloid cells to a pro-inflammatory phenotype, enhances MHC-II and CD86 expression, and synergizes with anti–PD-1 therapy to improve T cell infiltration and tumor regression (157, 158). In colorectal cancer (CRC), dual inhibition of LILRB1 and PD-L1 leads to enhanced dendritic cell activation, improved cross-presentation of tumor antigens, and greater expansion of tumor-specific CD8^+^ T cells, outperforming either monotherapy alone (157, 161). In acute myeloid leukemia (AML), LILRB2 is upregulated on leukemic stem cells and immunosuppressive myeloid cells. Blocking LILRB2, especially in conjunction with cytokine-based therapies like IL-15 superagonists, facilitates the clearance of leukemic cells by restoring innate immune activation and promoting NK and T cell responses (159). In hepatocellular carcinoma (HCC), LILRB1/LILRB2 suppress cytokine production by liver-infiltrating myeloid cells. Inhibition of these receptors, especially when combined with tumor vaccines or anti–PD-1, has been shown to increase IFN-γ and IL-12 production, supporting a pro-inflammatory tumor microenvironment (157, 161, 162). In melanoma, LILRB1 is enriched on tumor-infiltrating MDSCs and TAMs. Blocking LILRB1 signaling in combination with checkpoint blockade enhances cytotoxic T cell infiltration, remodels the tumor immune landscape, and delays tumor growth in preclinical models (163, 164).

In summary, targeting LILRBs, particularly LILRB1 and LILRB2,may help reprogram suppressive myeloid cells into a pro-inflammatory phenotype, enhance antigen presentation, and support the recruitment and activation of effector T cells. Inhibition of LILRB signaling may also synergize with anti–PD-1/PD-L1 therapies, tumor vaccines, or cytokine-based treatments to overcome immune resistance and broaden the efficacy of immunotherapy (157, 161, 163–165).

#### 4.2.2 Clinical trials on LILRB-targeted therapies

LILRB-targeted agents are advancing into clinical development based on strong preclinical evidence supporting their role in immune suppression and resistance to checkpoint inhibitors. Among the most advanced clinical-stage programs is IO-202, a humanized monoclonal antibody developed by Immune-Onc Therapeutics, which targets LILRB4. It is currently being tested in a Phase I clinical trial (NCT04372433) for acute myeloid leukemia (AML) and chronic myelomonocytic leukemia (CMML), both as monotherapy and in combination with nivolumab. Early data suggest that IO-202 is well tolerated and shows signs of immune activation, including enhanced T cell infiltration and pro-inflammatory cytokine profiles (162, 163). Another agent, NG-641, an oncolytic viral vector encoding an anti–LILRB2 antibody, is in early-phase clinical trials for patients with solid tumors, including NSCLC and colorectal cancer. This vector also expresses chemokines and immune-stimulatory molecules, aiming to remodel the tumor microenvironment and overcome resistance to PD-1 blockade. These trials represent a growing effort to leverage LILRB inhibition not only to directly relieve myeloid immunosuppression, but also to synergize with existing immunotherapies in cancers where monotherapy checkpoint inhibition has shown limited success (164, 165).

Several clinical challenges hinder the progress of LILRB-targeted therapies. One major obstacle is the broad expression and structural similarity of LILRB family members. Since LILRBs are involved in maintaining self-tolerance and immune homeostasis, their blockade may induce autoimmune or inflammatory toxicities, especially when combined with other immunotherapies such as PD-1 or CTLA-4 inhibitors. Another issue is the lack of robust predictive biomarkers to stratify patients likely to benefit from LILRB-targeted agents. As research progresses, biomarkers of LILRB expression, such as tumor-associated MHC-I/HLA-G levels or myeloid cell signatures, may help stratify patients most likely to benefit from LILRB-targeted therapy. However, More biomarkers for LILRB-targeted therapy are needed clinically. While LILRB1/2 overexpression has been observed in some cancers including AML, prostate, liver, and breast cancer, there is limited information on the functional heterogeneity of LILRB-expressing cells in the tumor microenvironment across patients. This heterogeneity contributes to variable therapeutic responses and complicates clinical trial design. Additionally, preclinical models of LILRB biology are limited, especially since LILRB signaling differs significantly between mice and humans. This translational gap poses difficulties for dosing, efficacy prediction, and safety assessment in early-phase trials. Lastly, early clinical data are sparse, and most compounds remain in preclinical or Phase I trials, with few published results on efficacy or toxicity, further delaying clinical adoption (162–165).

### 4.3 V-domain Ig suppressor of T cell activation

#### 4.3.1 VISTA as an immune checkpoint in myeloid cells

VISTA is a unique immune checkpoint molecule predominantly expressed on myeloid cells (166). VISTA binds to VSIG3 and PSGL-1 and regulates immune homeostasis, particularly under inflammatory or tumorigenic conditions (167). VISTA functions through mechanisms that suppress T cell proliferation, cytokine production, and antigen-specific activation, thereby contributing to immune tolerance (166, 167). In the TME, VISTA is highly expressed on immunosuppressive myeloid populations and also on T cells It exerts its effects by engaging receptors on T cells and other myeloid cells, delivering inhibitory signals that blunt effector function (167). Moreover, VISTA upregulation is often observed in tumors resistant to PD-1/PD-L1 blockade, suggesting it plays a compensatory role in maintaining immune suppression. This makes VISTA a critical checkpoint for regulating myeloid–T cell interactions and a promising target in T cell–excluded tumors (167, 168).

#### 4.3.2 Strategies to block VISTA signaling and enhance T cell responses

Therapeutic strategies aimed at blocking VISTA signaling seek to reprogram the suppressive myeloid compartment and restore T cell function within the TME. These approaches include (1):. Monoclonal antibodies that block VISTA’s interaction with its ligands or receptors, thereby lifting the suppressive signals on T cells (169) (2);. Bispecific antibodies targeting VISTA alongside other checkpoints to simultaneously engage multiple arms of immune suppression (170) (3);. Antagonistic antibodies that bind VISTA on antigen-presenting cells to reprogram them toward a pro-inflammatory phenotype, boosting antigen presentation and co-stimulation (171).

In non-small cell lung cancer (NSCLC), VISTA is highly expressed on myeloid-derived suppressor cells (MDSCs) and regulatory T cells (Tregs) within the tumor microenvironment (TME), contributing to immune suppression and resistance to PD-1 blockade. Preclinical studies demonstrate that VISTA inhibition significantly increases CD8^+^ T cell infiltration, enhances IFN-γ and TNF-α secretion, and restores anti-tumor immunity, particularly when combined with anti–PD-1 therapy in PD-1-resistant models (172). In melanoma, VISTA expression rises following PD-1/CTLA-4 blockade, suggesting a compensatory upregulation. Combining VISTA inhibitors with anti–CTLA-4 or anti–PD-1 antibodies overcomes adaptive resistance mechanisms and leads to robust tumor regression in murine melanoma models by reducing Treg frequencies and increasing granzyme B+ cytotoxic T lymphocytes in the tumor (171, 173). In pancreatic ductal adenocarcinoma (PDAC), an immunologically “cold” tumor, VISTA is highly expressed on infiltrating macrophages and MDSCs. VISTA blockade reprograms macrophages to an M1-like phenotype and allows for immune priming, which synergizes with PD-L1 inhibition to promote T cell–mediated tumor control in preclinical PDAC models (169, 170, 173). In prostate cancer, VISTA expression on TAMs correlates with poor T cell infiltration and immune exclusion. Targeting VISTA, especially in combination with cytokine-based treatments (e.g., IL-12 or IFN-α), reduces suppressive myeloid cell activity and enhances responsiveness to checkpoint inhibitors (174). In glioblastoma, VISTA is enriched in myeloid cells and contributes to the highly immunosuppressive TME. Preclinical evidence suggests that VISTA blockade improves antigen presentation and T cell infiltration and works synergistically with PD-1 blockade to overcome resistance in this typically refractory cancer (169, 170, 173).

In summary, Blocking VISTA has been shown in preclinical models to enhance CD8^+^ T cell infiltration, increase pro-inflammatory cytokine production, and reduce Treg and MDSC activity (173). Importantly, VISTA inhibition can work synergistically with PD-1/PD-L1 or CTLA-4 blockade, overcoming resistance mechanisms in immunotherapy-refractory tumors (169, 173).

#### 4.3.3 Progress in clinical development of anti-VISTA agents

The promising preclinical data supporting VISTA as a myeloid checkpoint has led to the advancement of several anti-VISTA therapies into clinical trials. Among the leading candidates is CA-170, an oral small-molecule antagonist targeting both VISTA and PD-L1, developed by Curis, Inc. CA-170 entered phase I clinical trials in patients with advanced solid tumors and lymphomas and showed a favorable safety profile, though efficacy data remains preliminary (173). Another notable agent is JNJ-61610588 (CI-8993), a humanized monoclonal antibody against VISTA developed by Janssen, currently being tested in early-phase clinical trials (e.g., NCT04475523) for advanced cancers (172, 174, 175).

While anti-VISTA therapies are still in the early clinical development stage, they hold great promise, especially in tumors with high myeloid infiltration and resistance to traditional checkpoint inhibitors (170–173, 176, 177). However, its clinical translation still faces several key challenges. One major issue is context-dependent expression. VISTA is predominantly expressed on myeloid cells and naïve T cells, and its expression can dynamically shift depending on tumor type, stage, and microenvironmental cues. This makes patient selection and biomarker development particularly difficult. Moreover, the mechanisms of action of VISTA are still incompletely understood. Its immunosuppressive roles are not always redundant with other checkpoints like PD-1 or CTLA-4. Consequently, designing combination therapies that avoid excessive immune activation while providing synergy remains challenging. From a safety standpoint, early clinical trials of VISTA inhibitors, including CA-170, have shown manageable toxicity profiles, potentially leading to autoimmune or inflammatory adverse events. Lastly, the lack of robust pharmacodynamic markers to assess target engagement and immune modulation in patients has slowed trial progress. Many ongoing trials remain in early phases, and evidence of clinical efficacy is still limited, necessitating more mechanistic studies and refined patient stratification strategies. As result, ongoing trials assess not only safety and pharmacodynamics but also biomarker-driven patient stratification, aiming to identify populations most likely to benefit from VISTA blockade (170–177).

### 4.4 Cluster of differentiation 40

#### 4.4.1 Role of CD40 in myeloid cell activation and anti-tumor immunity

CD40 is a member of the tumor necrosis factor receptor (TNFR) superfamily and is expressed on a variety of antigen-presenting cells (APCs) (178). Its ligand, CD40L (CD154), is primarily expressed on activated CD4^+^ T cells (179). Engagement of CD40 on myeloid cells triggers a cascade of intracellular signaling events involving NF-κB, MAPK, and PI3K/AKT pathways, leading to upregulation of co-stimulatory molecules, cytokine secretion, and enhanced antigen presentation (179–181). In the TME, CD40 signaling reprograms TAMs and dendritic cells from a suppressive, tolerogenic state to an inflammatory, immunostimulatory phenotype. This shift supports the recruitment and activation of effector T cells, especially CD8^+^ cytotoxic T lymphocytes, and promotes tumor cell killing through both direct and indirect mechanisms (182, 183).

#### 4.4.2 CD40 agonists in myeloid cell targeting: effects and clinical potential

CD40 agonists are a class of immunotherapeutic agents designed to mimic CD40L, which activate CD40-expressing myeloid cells within the TME. These agents include agonistic monoclonal antibodies (e.g., selicrelumab (184), APX005M (185), CDX-1140 (186)) and CD40L fusion proteins, which engage CD40 and promote pro-inflammatory remodeling of the TME (187). Preclinical studies have shown that CD40 agonists can eradicate established tumors, even in the absence of checkpoint blockade, by bridging innate and adaptive immunity (188).

In pancreatic ductal adenocarcinoma (PDAC), CD40 agonists have demonstrated the ability to reprogram tumor-associated macrophages (TAMs) into tumoricidal cells that facilitate stromal remodeling and promote T cell–independent tumor regression. In a landmark mouse model study, CD40 agonist therapy alone induced substantial tumor shrinkage without requiring PD-1 or CTLA-4 checkpoint inhibition, highlighting a potent innate immune mechanism (180). In melanoma, CD40 activation enhances dendritic cell (DC) maturation and cross-presentation of tumor antigens, leading to robust CD8^+^ T cell–mediated cytotoxicity. These effects persist even in the absence of additional checkpoint modulation, underscoring CD40’s ability to prime adaptive immunity (181–183). In bladder cancer, preclinical studies using ADC-1013, a human CD40 agonistic antibody, have shown eradication of subcutaneous tumors and development of long-term protective memory responses. These effects were CD8^+^ T cell–dependent but did not require co-administration of checkpoint inhibitors (181–183). In lymphoma, CD40 ligation on malignant B cells leads directly to apoptosis and enhances their immunogenicity, triggering cytotoxic T cell responses capable of eradicating disease. These outcomes occurred independently of PD-1 or CTLA-4 blockade, making CD40 agonists a promising standalone immunotherapy for hematologic malignancies (181). In colorectal cancer, CD40 agonists have been shown to improve antigen presentation by tumor-resident DCs and promote T cell infiltration and expansion within the tumor microenvironment (182, 183). This bridging of innate and adaptive arms led to tumor regression even without additional immune checkpoint therapies. In non–small cell lung cancer (NSCLC) and breast cancer, CD40 stimulation has been associated with enhanced macrophage activation and expansion of effector T cells in murine models, contributing to tumor control (184, 188).

In clinical trials, CD40 agonists have demonstrated tolerable safety profiles, with some showing partial responses and disease stabilization in patients with pancreatic cancer, melanoma, and non-small cell lung cancer (NSCLC) (189–192). In pancreatic ductal adenocarcinoma (PDAC), CD40 agonist therapy—particularly when combined with chemotherapy—has demonstrated disease stabilization and improved immune cell infiltration, as observed in early-phase trials using APX005M and selicrelumab (189). In melanoma, agents such as CP-870,893 have induced partial responses and enhanced T cell activation in patients, with manageable infusion-related adverse events (190, 191). In non–small cell lung cancer (NSCLC), CD40 agonists used as monotherapy or in combination with immune checkpoint inhibitors have led to stable disease in a subset of patients, while enhancing dendritic cell function and T cell priming (190, 191). These findings support the rationale for integrating CD40 agonists into combination regimens to potentiate anti-tumor immunity in immunologically cold tumors.

CD40 agonists have shown promise in enhancing anti-tumor immunity by activating antigen-presenting cells and bridging innate and adaptive immune responses. However, their clinical development has been limited by safety concerns, especially related to cytokine release syndrome (CRS). Systemic CD40 activation can lead to excessive production of pro-inflammatory cytokines (e.g., TNF-α, IL-6), causing flu-like symptoms, hypotension, and liver enzyme elevations, even at low doses (192, 193). Another challenge lies in achieving the right balance of immune activation without triggering off-target inflammation. Because CD40 is broadly expressed on immune and non-immune cells systemic delivery of agonistic antibodies may result in non-specific tissue toxicity, especially in the liver and spleen (180, 194, 195).Furthermore, not all patients respond to CD40 agonists, and responses are often transient. The lack of reliable predictive biomarkers makes it difficult to select appropriate patients or monitor therapeutic response in real time (192). Finally, CD40 agonists may not work well as monotherapies in many solid tumors and often require combination with chemotherapy or checkpoint blockade to achieve meaningful efficacy (180, 192–195).

#### 4.4.3 Combination therapies involving CD40 agonists

Given the broad immunostimulatory effects of CD40 agonists, their use in combination therapies is a major area of clinical exploration. One of the most promising strategies is combining CD40 agonists with immune checkpoint inhibitors (195–197). CD40 activation primes and expands T cell responses, while checkpoint blockade sustains T cell activity by preventing exhaustion (195–197).CD40 agonists are also being tested in combination with chemotherapy, which can provide a source of tumor-associated antigens (TAAs) via immunogenic cell death and enhance antigen cross-presentation (198). Other combinations under investigation include CD40 agonists with radiation therapy, TLR agonists, STING activators, or cancer vaccines, all of which can further boost antigen release and immune activation (198–201). Ultimately, the goal is to develop multi-pronged immunotherapeutic regimens where CD40 agonism serves as the immune ignition switch, enabling and enhancing the efficacy of co-administered treatments.

## 5 Conclusion

Recent research has highlighted the critical role of myeloid cells—particularly TAMs and MDSCs—in promoting tumor growth and suppressing immune responses. These cells create an immunosuppressive tumor microenvironment by inhibiting T cell activity, releasing anti-inflammatory cytokines, and supporting tumor angiogenesis and metastasis. Strategies to target these cells include depletion (e.g., CSF1R inhibitors), functional reprogramming (e.g., CD40 agonists, PI3Kγ inhibitors), and blocking their recruitment. These approaches aim to reduce immunosuppression and restore the ability of the immune system to recognize and destroy cancer cells.Moreover, combining myeloid cell-targeting therapies with existing treatments—such as immune checkpoint inhibitors, chemotherapy, or radiotherapy—has shown promising synergistic effects. Targeting MDSCs and reprogramming TAMs can enhance the effectiveness of immunotherapies by reversing resistance and promoting stronger, more durable immune responses. Overall, myeloid cell-targeting has emerged as a powerful strategy to reshape the tumor microenvironment and support more effective, personalized cancer treatments.

Targeting myeloid cells—such as TAMs and MDSCs—is a promising approach to enhance cancer immunotherapy. In the future, therapies are expected to become more selective and precise, using advanced delivery systems like nanoparticles and cell-specific antibodies to modulate these cells without harming healthy immune function. Researchers are also developing drugs that reprogram suppressive myeloid cells into pro-inflammatory, anti-tumor cells. This shift may allow for more durable responses, especially when combined with immune checkpoint inhibitors, chemotherapy, or radiation.

Looking ahead, personalized medicine will play a major role in guiding treatment choices. Biomarkers and tumor profiling techniques—such as single-cell sequencing and spatial transcriptomics—will help identify patients whose tumors rely heavily on myeloid-driven immunosuppression. In addition, combining myeloid-targeting strategies with other novel approaches, like cancer vaccines or CAR-T cell therapy, could create multi-layered treatments that better overcome tumor resistance. Together, these future strategies aim to make cancer immunotherapy more effective, personalized, and widely applicable.

Myeloid cell-targeting therapies have shown growing potential to improve clinical outcomes in cancer patients by overcoming tumor-induced immune suppression. TAMs and MDSCs are often linked to poor prognosis, as they help tumors evade the immune system. By reprogramming these cells or reducing their numbers, researchers aim to restore the body’s natural anti-tumor immune responses. The goal of these strategies is to improve overall survival and long-term disease control. As personalized medicine advances, myeloid-targeted therapies may be matched more precisely to patients whose tumors rely heavily on myeloid-driven suppression. Clinical trials are ongoing to evaluate these approaches across different cancer types, and early data are promising. If successful, these therapies could significantly extend survival for patients who currently have limited treatment options, offering a new way to enhance the success of modern cancer immunotherapy.

While numerous preclinical studies and early-phase clinical trials have demonstrated the promise of myeloid-targeted therapies, translating these findings into effective and broadly applicable cancer treatments remains challenging. One major barrier is the heterogeneity and plasticity of myeloid cells in the tumor microenvironment, which complicates both therapeutic targeting and biomarker development. In addition, compensatory pathways, such as upregulation of alternative immunosuppressive checkpoints, can limit the durability of responses. Clinically, limited efficacy of monotherapy in late-stage trials and immune-related toxicities pose challenges in dosing and patient selection. From a regulatory standpoint, the lack of validated biomarkers and standardized assays impedes trial design and approval. To address these issues, future strategies should focus on combination regimens, real-time immune monitoring, and patient stratification using multi-omics approaches to tailor therapy more effectively.

In clinical studies biomarker development can help in understanding how myeloid-targeted therapies can be personalized for cancer patients. Currently, several candidate biomarkers are under investigation to predict response to therapies targeting tumor-associated macrophages (TAMs), myeloid-derived suppressor cells (MDSCs), and dendritic cells. For example, high baseline expression of CSF1R or its ligands (CSF1, IL-34) in the tumor microenvironment has been associated with better responses to CSF1R inhibitors. Other promising directions include TREM2 expression, which correlates with myeloid-mediated immune exclusion and resistance to PD-1 inhibitors, and VISTA or LILRB2 upregulation, which may predict a suppressive myeloid phenotype. Advances in spatial transcriptomics and single-cell RNA-seq have further enabled the identification of myeloid cell states that may respond differentially to therapies. Moreover, dynamic biomarkers such as circulating cytokines (e.g., IL-6, TNF-α) and soluble checkpoint molecules are being explored as early indicators of treatment response or toxicity. Despite these advances, most of these biomarkers remain exploratory and require validation in larger clinical cohorts. Despite significant interest in personalizing myeloid-targeted cancer therapies, biomarker development remains limited by several challenges. A major issue is the high heterogeneity and plasticity of myeloid populations, which makes it difficult to identify stable, predictive markers. Furthermore, many candidate biomarkers including CSF1R, PI3Kγ, and TREM2, exhibit dynamic and context-dependent expression, complicating their use for patient stratification. The lack of standardized and validated assays across clinical studies hinders reproducibility and translation. Finally, few biomarkers have undergone prospective validation in large-scale trials, and most remain exploratory, often limited to preclinical or early-phase settings. As a result, incorporating biomarker-driven patient selection and pharmacodynamic monitoring into clinical trials will be key to realizing the full potential of myeloid-targeted immunotherapies and improving treatment outcomes.
